# Adopting New International Health Instruments – What Can We Learn From the FCTC? Comment on "The Legal Strength of International Health Instruments - What It Brings to Global Health Governance?"

**DOI:** 10.15171/ijhpm.2017.78

**Published:** 2017-07-15

**Authors:** Marlies Hesselman, Brigit Toebes

**Affiliations:** ^1^Department of International Law/Global Health Law Groningen, Faculty of Law, University of Groningen, Groningen, The Netherlands.; ^2^Global Health Law Groningen Research Centre, Faculty of Law, University of Groningen, Groningen, The Netherlands.

**Keywords:** Framework Convention on Tobacco Control (FCTC), Non-communicable Diseases (NCDs), Law and Policy Solutions, International Relations Theory, Unhealthy Diets

## Abstract

This Commentary forms a response to Nikogosian’s and Kickbusch’s forward-looking perspective about the legal strength of international health instruments. Building on their arguments, in this commentary we consider what we can learn from the Framework Convention on Tobacco Control (FCTC) for the adoption of new legal international health instruments.

## Introduction

With the adoption of the World Health Organization (WHO) Constitution in 1946, the World Health Assembly (WHA) was granted considerable standard setting powers, including the authority to adopt binding treaties and regulations.^1^ During the first 50 years of its existence, the WHA barely made use of this possibility: the only legally binding instrument it adopted were the International Health Regulations,^2^ offering a protection mechanism against the spread of infectious diseases. Yet, as Nikogosian and Kickbusch^3^ point out in their perspective to this journal, the start of the 21st century opened a new phase where legal instruments were more readily adopted. During these years a revised set of the International Health Regulations was adopted as well as the first WHO treaties: the Framework Convention on Tobacco Control (FCTC, 2003) and its first Protocol (2013).^4-6^

**Figure 1:**
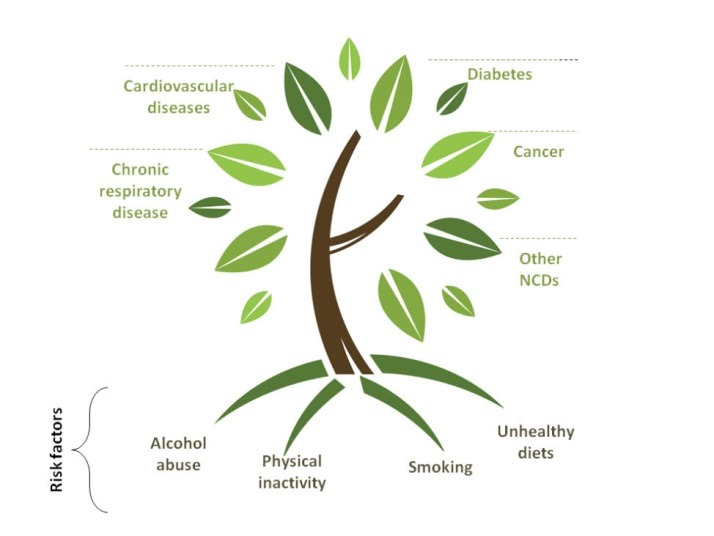
Modifiable Behavioural Risk Factors (Regional Office for the Eastern Mediterranean, WHO). Reprinted with permission from the WHO.

The present authors take inspiration from the FCTC in asking whether further instruments in the area of (chronic) non-communicable diseases (NCDs) may be possible, for we consider curbing NCDs the highest priority in global health. It is widely recognised that the majority of deaths presently occurring globally are the result of NCDs – in particular cardiovascular diseases, most cancers, chronic respiratory diseases and diabetes. In relation to this, it is also broadly acknowledged that NCDs are fuelled by the persistent increase in NCD-related risk factors, ie, smoking, excess alcohol use, unhealthy eating and physical inactivity.^7^ However, the only risk factor that is currently regulated through a treaty is tobacco, through the FCTC (Figure 1).

As Nikogosian and Kickbusch explain, the FCTC and its Protocol “unlocked” the treaty-making power of WHO contained in its Constitution, and they mention various successes of the FCTC as the first legally binding WHO instrument, dedicated to NCDs. They also recognize that “further research in the coming years would bring more details and depths to the topic.”

This commentary responds to the authors’ viewpoints by reflecting on the opportunities for adopting new international health standards on NCDs within the context of the WHO. In our view, the successful legal standard setting through the FCTC raises at least two important further questions: (1) what does it take to adopt new (binding) standard-setting instruments for NCDs; and (2) what should be the nature, scope and substance of these instruments?

## What Does it Take for New International Health Instruments to be Adopted?

As submitted by Nikogosian and Kickbusch, “the very fact that globally binding instruments in health were feasible” kick-started debates and expectations for further possible instruments. Examples of such instruments suggested so far, include a Framework Convention on Pharmaceutical Innovation and a broader Framework Convention of Global Health.^8,9^ Another example under serious, but slow, consideration within the WHO, is a Convention on Research and Development.^10^

At the end of their article, the authors express “little doubt that the international health instrumentarium will further expand and develop, with binding and soft-law instruments alike.” We do not disagree with this statement, but want to put into the mix that, in order to arrive at legal standards, or in accelerating standard-setting processes, it will be highly instrumental to better understand what drives or accommodates the development of new international (treaty) norms.

**Figure 2:**
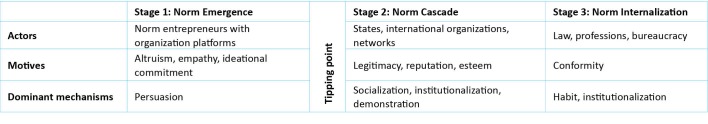
Original Norm Life-Cycle Model by Sikkink and Finnemore.^11^

One way of understanding the dynamics and conditions of new norm creation is offered by so-called norm life-cycle models, developed in social constructivist strands of international relations theory.^11^ According to such models, the creation of new (treaty) norms tends to follow fairly straight-forward paths or ‘life-cycles,’ and may require certain actors and conditions to come together throughout subsequent stages of norm creation (the original ‘norm-life cycle’ model is by Finnemore and Sikkink) (Figure 2).^11^

Refinements of the original model formulate in more detail the various stages of norm development, *inter alia* proposing that new (treaty) norms follow a ‘seven-step sequence,’ starting with (*i*) the formulation of an initial idea by an (influential) (group of) norm-entrepreneur(s) with a strong organizational platform to spread their message.^12^ Although different people and groups can be norm entrepreneurs, including, importantly, NGOs, in the context of the FCTC, a common understanding is that Gro-Harlem Brundtland, the former Director-General of the WHO, was an instrumental norm entrepreneur in persuading the WHO to move forward on the FCTC.^13^ In contrast, Margaret Chan seems to have suggested in 2012 on the R&D Convention that non-binding options could also be explored, which at least some commentators count as an important reason for failure to move forward at that time.^10^

Typically, formulation of initial ideas is followed by (*ii*) ‘network configuration’ and (*iii*) ‘issue formulation,’ as essential components of the norm emergence process. Issue formulation entails defining the ‘technical and normative scope’ of the problem, and the wording of the newly proposed norm. ‘Network configuration’ implies building a network of supporters that can lend support, and gives the norm legitimacy and credibility.^12^ This can entail wider civil society support, or institutional support by key actors. On these factors, a recent study contends that global alcohol control advocacy networks have been less successful in advancing new norms in comparison to tobacco control networks, because alcohol advocacy yet has ‘to overcome divisions based on competing problem definitions and solutions to alcohol harm.’^14^ Moreover tobacco control networks appear to have effectively evolved ‘from a group of dedicated individuals to a global coalition of membership-based organizations’ and ‘more effective in creating and maintaining wide-spread consensus about effective policies to harm reduction.’^14^ Norm-cycle theory highlights that achieving ‘scientific closure,’ along with ‘normative closure,’ on the norm, are essential components to norm emergence.^12^ Naturally, this can be challenging for certain global health challenges, for industries may actively seek to prevent or delay scientific closure on the harmful nature of the use of their products. Finally, concluding stages of norm adoption would include (*iv*) dialogues between proactive and conservative states, and (*v*) a moment of ‘political’ closure.^15^ Political closure presupposes a ‘critical mass of relevant [opposing and supporting] State actors’ adopting the new norm, and is typically referred to as the ‘tipping point’ of a norm. This is the moment after which stages of (*vi*) (soft or hard) ‘legalization’ and (*vii*) institutionalization and operationalization come in sight.

While it is impossible, in this short article, to assess how various current proposals for new instruments hold up against this model, looking ahead at possible lessons that could be gleaned from the FCTC through such models, are, first: who are the current norm entrepreneurs driving new (binding) norms on NCD issue areas, and what are their organizational platforms? To what extent are nascent international NCD coalitions separately or jointly formulating a clear message on salient issues? Additional issues include: how has the FCTC process been influenced by particular norm entrepreneurs, solid network configuration, issue formulation, and a measure of ‘scientific closure’ on the harmful nature of tobacco use?^14^ While we do not purport to have all the answers in this Response, these important questions deserve to be raised.

## Nature, Scope and Substance of New International Health Instruments

Alongside an identification of the dynamics, processes and conditions necessary for moving towards the adoption of new standards, we should identify what these instruments should regulate as a priority issue, and what should be their nature, scope and content. As mentioned, currently tobacco is the only risk factor regulated through a treaty. The other mentioned risk factors are currently addressed through the non-binding Global Strategy on Diet, Physical Activity and Health and the Global Strategy to Reduce the Harmful Use of Alcohol.^16,17^ A recent innovation is the new target-setting ‘WHO Global Action Plan for the Prevention and Control of NCDs 2013-2020.’ This instrument formulates nine global targets on the four NCD risk factors.

It is of crucial importance to study the impact and effectiveness of existing strategies, and to consider whether a legally binding instrument would be more effective. Nikogosian and Kickbusch list a number of benefits of binding health instruments, as opposed to non-binding ones. Yet, the current reality in international law is, that against the trend set out by Nikogosian and Kickbusch in their article, treaties are increasingly being superseded by ‘informal lawmaking,’ involving new forms, processes and actors for norm creation.^18^ These can include Guidelines, Codes, Standards or even Action Plans, including the ones already mentioned above.^18^

Indeed, while treaties are authoritative and influential, and can play an important role in domestic legal systems, it is important to consider a broad range of regulatory options, including both formal and informal ones, when it comes to designing new NCD instruments. In fact, contrary to some popular views, States may opt for non-binding instruments not necessarily to escape being legally bound, but because the community as a whole favours swift action and responses, or greater multi-stakeholder involvement, which highly formalized, binding, technical treaty negotiations, and possibly lengthy ratification procedures, do not always allow for.^18^ Again, this reason has been cited in the context of States not being eager to move towards a binding treaty on R&D in 2012.^10^ In this respect, further research on when binding treaty adoption may be pursued in favour of, or alongside, non-binding instruments is welcome too.

Finally, we may need to consider the manner in which international norm adoption can be successfully informed or driven by domestic laws and successes. For example, there is clear evidence from Mexico that taxes on sugar-sweetened beverages result in proportional reductions in consumption, especially if aimed at raising the retail price by 20% or more.^19^ This evidence could inspire provisions on taxes in a possible treaty regulating unhealthy diets. While domestic good practice examples cannot simply be transplanted to the international level, they can provide useful examples and elements for new international health instruments. In particular, the further experimentation with and implementation of the ‘menu of policy options’ currently listed in the new WHO NCD 2013-2030 Action Plan can yield further building blocks.

All in all, we clearly see a window of opportunity to consider and pursue the adoption of new standard-setting instruments in the field of health. What is needed to start with, is a group of vocal norm entrepreneurs in practice and academics, with a strong and concerted message, along with optimism, creativity and commitment from within the WHO to follow through on its law-making mandate, and for norm leaders to stand up in this context. The sheer size of the NCD pandemic demands a forward-looking perspective and the most progressive approach possible.

## Ethical issues

Not applicable.

## Competing interests

Both authors declare that they have no competing interests.

## Authors’ contributions

Both authors contributed to comment equally on the basis of earlier collaboration. BT: set up main outline and drafted paragraphs dealing with WHO/health law and NCDs. MH: drafted paragraphs on norm-cycle dynamics/emergence new instruments and ensured final synergy of various parts of the comment. Both authors read and agreed final content.

## Authors’ affiliations

^1^Department of International Law/Global Health Law Groningen, Faculty of Law, University of Groningen, Groningen, The Netherlands. ^2^Global Health Law Groningen Research Centre, Faculty of Law, University of Groningen, Groningen, The Netherlands.
